# Mental Health Levels and Incidence of Musculoskeletal Complaints among Speed Boat Crew Members

**DOI:** 10.5812/traumamon.8177

**Published:** 2013-01-15

**Authors:** Farzaneh Zigheimat, Abbas Ebadi, Fatemeh Rahmati Najarkolaei, Mohammad Malakoti, Farhad Kheiri Tootkaleh

**Affiliations:** 1 Department of Community Health Nursing, Faculty of Nursing, Baqiyatallah University of Medical Sciences, Tehran, IR Iran; 2 Health Research Center, Baqiyatallah University of Medical Sciences, Tehran, IR Iran; 3 Faculty of Medicine, Tehran University of Medical Sciences, Tehran, IR Iran

**Keywords:** Mental Health, Musculoskeletal, Crews of Express Speed Boats

## Abstract

**Background:**

The occupational health is an important issue. In some jobs, the working conditions contribute to musculoskeletal complaints and the overall health of the individual is compromised. Musculoskeletal complaints have gained credence in the public as one of the most important problems in the field of occupational diseases. Physical and mental health of crew members with critical jobs and stressful environments must be considered as well.

**Objectives:**

This study performed an assessment on levels of mental health and the correlation with the frequency of accompanying musculoskeletal complaints (such as neck, back and knee pain) of crew members of speed boats.

**Material and Methods:**

149 onboard crew members of speed boats were recruited in a descriptive-correlation study by nonrandom sampling using conducted GHQ12, NMQ and demographic questionnaires.

**Results:**

Although 63.8% (95 people) had what is conventionally defined as normal mental health, 36.2% (54 cases) had an inherent mental health condition. Overall, 61.1% (91 cases) suffered from back pain, 60.4% (90 cases) complained of knee pain, and 40.3% (60 patients) complained of neck pain. The combination of knee and back pain (48.3%) were the most common complaints whereas the combination of neck and knee pain (31.5%) were the least frequent; 28.2% complained of pain in all three areas. Interestingly, there was correlation between the presence of musculoskeletal complaints and less than optimum mental health.

**Conclusions:**

Due to the high number of musculoskeletal complaints and the compromised mental health conditions among one-third of the onboard crew members of speed boats, attention for maintaining and improving the health of these members must be considered.

## 1. Background

The World Health Organization (WHO) defines health as a state of complete physical, mental and social well-being and not merely the absence of disease and disability. This statement has been augmented in recent years to include a "dynamic social and economic life". The physical dimension of health is more visible; more easily assessed and more likely to be understood. Physical stability means "complete function" of the body (1). However, recently researchers discovered that psychological factors can cause mental illness, and manifest in the contexts of other diseases (2).

Occupational health is one of the most important and significant aspects of health. When work is perfectly consistent with the goals, capabilities and human limitations of a person, it plays a major role in promoting mental and physical health (3). Musculoskeletal disorders (MSDs) are defined as injuries and disorders of the muscle, nerves, tendons, ligament, joints, cartilage and spinal disc (4). The main causes of MSDs are due to awkward positions (50.2%), strenuous forces (28.2%), and repetitive work (16.8%) (3). Musculoskeletal complaints are one of the most important occupational health issues in today's world and are prevalent in many jobs (5). Musculoskeletal complaints are the second largest health problems in the world and nearly two-thirds of them are due to work-related diseases and disorders. MSD’s are only secondary to the frequent traumas suffered on a worldwide basis.

The physical and mental health of workers is threatened due to various events and chronic occupational stress (6). Occupational stresses can cause many psychological problems such as nervous, emotional and mental stresses; in addition, it can cause physical problems such as cardiovascular disease, muscular and skeletal pain, pulmonary and gastrointestinal disorders and also decrease the quality of one’s overall function (7). Depending on the type of job and work environment, their effects can damage the muscles, nerves, tendons, ligaments, joints, cartilage and spine. Such complaints may be caused by prolonged exposure to risk factors (mechanical stress, lack of proper ergonomic work, etc.), occur gradually or suddenly as part of a long process or occur due to trauma to a part of the musculoskeletal system (8). It has been proven that the occupational related musculoskeletal disorders are associated with multiple occupational risk factors. These risk factors comprise environmental physical factors, (such as pressure, posture, shipping and handling objects, vibration) and psychosocial stress factors (9).

Personal factors such as age, sex, anthropometric characteristics and physical agents in the workplace including: heat, coolness, humidity, air conditioning, heat radiation, noise, vibration and ionizing radiation etc. may have a negative impact on health. These factors, alone or in combination with each other, can affect the health and performance of workers (10). Also, musculoskeletal complaints have been linked with occupational stress in some jobs such as nursing. (11).

## 2. Objectives

This study was undertaken to evaluate the mental health and its relationship with the incidence of musculoskeletal complaints (neck, back and knees pain) among the crew of express speed boats.

## 3. Materials and Methods

In a correlation-descriptive study that was conducted from April to November in 2010, 132 participants of speed boats were assessed. The inclusion criteria were at least one year of working experience on a speed boat. Easy and availability random sampling was performed.

### 3.1. Data Collection

Three questionnaires were employed to gather the data as the following:

1. Demographic questionnaire to determine demographical features.

2. Goldberg et al. standard mental health questionnaire (1997), the 12 questions (GHQ12) to determine the level of mental health.

3. Nordic standard questionnaire (NMQ) to investigate the spread of musculoskeletal complaints among the crew.

### 3.2. Statistical Analysis

Face-to-face interviews were conducted with 149 crew members and the questionnaires were analyzed by SPSS version 16 using independent t-test and Chi-Square.

## 4. Results

The mean age of the 149 participants was 28 with the range being 19-45 years-old; 103 subjects (69.1%) were married. The mean of BMI was 23.7 and the mean job experience was 7.4 years. The mean of working experience on an express floating vessel was 5.6 years however the mean expenditure of time on an express speed boat was 42 hours per week.

According to the findings, 54 subjects (36.2%) had a mental health condition whereas 95 subjects (63.8%) had normal mental health. The distribution of absolute and relative frequencies of the crew’s mental health was on the basis of marital status, their working shift, their position and their duty as revealed in Table 1. The level of mental health of the crew with regard to their demographics is presented in Table 2.

**Table 1:** Distribution of Mental Health of Speed Boat Crews on the Basis of Marital Status, Work Shift, Position and Duty

General health Features	Normal	Abnormal	Total
**Marital status, No (%)**			
Single	29 (30.5)	17 (31.5)	46 (30.9)
Married	66 (69.5)	37 (68.5)	103 (69.1)
**Work shift**			
Official (fixed)	31 (32.6)	17 (31.5)	48 (32.2)
Unfixed	64 (67.4)	37 (68.5)	101 (67.8)
**Position**			
Upright	65 (68.4)	40 (74.1)	105 (70.5)
Seated	30 (31.6)	14 (25.9)	44 (29.5)
**Duty**			
Helmsman	63 (66.3)	33 (61.1)	96 (64.4)
Back-up	16 (16.8)	6 (11.1)	22 (14.8)
Repairman	9 (9.5)	10 (18.5)	19 (12.8)
Electrician	7 (7.4)	5 (9.3)	12 (8.1)

**Table 2:** The Correlation Between the Level of the Crew’s Mental Health and Demographic Features

	Age, Mean ± SD	Height, Mean ± SD	Weight, Mean ± SD	BMI, Mean ± SD	Working experience on floating vessels, Mean ± SD	Total working experience, Mean ± SD	Time spending on speed boat per week, Mean ± SD
**Normal **	27.1 ± 5.5	173 ± 5.3	69.6 ± 9.5	23 ± 2.9	4.9 ± 4.5	6.3 ± 5.6	39 ± 21.7
**Abnormal **	29.6 ±5.9	175 ± 6.3	76.3 ±8.3	24 ±2	6.9 ± 5.1	9.3 ± 6.6	47 ± 28.3
**Independent-t test**	0.01	0.08	0.001	0.001	0.001	0.004	0.03

The presence of neck, back and knee pains among the 149 subjects of speed boats during the last 12 months revealed that the most frequently reported case of pain and complications was the back in 91 subjects (61.1%); knee pain was the second most reported in 90 subjects (60.4%) and neck pain was the third most reported in 60 subjects (40.2%)(See Table 3). There was a significant statistical relationship among: a) neck pain and age, working experience on speed boat and the total job experience; b) backache and age, height, weight and the total working experience on speed boat, and c) pain in knees and the total job experience and time spent on the speed boat per week.

**Table 3:** Distribution Frequency of Musculoskeletal Complaints of Speed Boat Crew members in Neck, Back and Knees

Complaint Zone	Yes, no (%)	No, no (%)	Total, no (%)
**Neck**	60 (40.3)	89 (57.7)	149 (100)
**Back**	91 (61.1)	58 (38.9)	149 (100)
**Knee**	90 (60.4)	59 (39.6)	149 (100)

The Chi-Square indicated a statistically significant correlation between mental health and pain in the three zones (i.e. neck, back and knees, P < .01, Table 4). The complaint of both back and knee pain was reported in 48.3% (72 subjects), both neck and back in 31.5% (47 subjects), and both neck and knees in 34.5% (51 subjects) of crew members. Complaints of back, knee and neck pain at same time was reported by 28.2% (42 people). In this study, the mean age of the participants who had normal mental health was 27.2 years, and those who had mental health problems were 29.7 years. A significant correlation between age and mental health was found; health deteriorated with increased age (P < .01).

**Table 4:** Comparison of Mental Health Status and Musculoskeletal Problems

Mental health Painful zone	Normal, no (%)	Abnormal, no (%)	P value
**Neck**			P = 0.01
Yes	31 (32.6)	29 (53.7)	
No	64 (67.4)	25 (46.3)	
**Back**			P = 0.001
Yes	47 (49.5)	44 (81.5)	
No	48 (50.5)	10 (18.5)	
**Knee**			P = 0.001
Yes	51 (53.7)	39 (72.2)	
No	44 (46.3)	15 (27.8)	

The minimum weight of the subjects was 50, maximum was 98 and the mean was 72 kilograms. Of these, 95 subjects with mean weight of 69 kg had desired mental health, and 54 subjects with mean weight of 76 kg had undesired mental health. Statistical tests showed significant differences between mental health and weight, i.e., mental health decreased as weight increased (P < 0.000).

 The mean body mass index of 95 participants was 23, who were in desired health, and the mean body mass index of 54 participants was 24.8, who were in undesired health. Analyses showed a statistically significant relationship between BMI and overall health. Mental health of the subjects decreased with increasing body mass index (P < 0.000). Statistical tests showed a significant association between mental health and the work experience, i.e., hours worked on speed boat per week (P < 0.000).

## 5. Discussion

The physical and mental health of crew members is very important for appropriate performance especially under high pressure conditions where life and death are literally at stake. Due to the very high speed and maneuverability of speed boats, and the potentially overwhelming effect of sea waves and the dynamic pressure, these boats can result in threats to the health of the crew members; this study assessed the relationship between mental health and incidence of musculoskeletal complaints (neck, back and knee pain) among the crew. Based on the findings of this study, more than a third of the participants in the study have undesired (suspected) mental health attributes. These findings are similar to passenger driver (36.2%) (12).

In this study, the highest incidence of musculoskeletal complaints was allocated to the lower back pain (61.1%), and in the second place, knee pain (60.4%) and third, (40.3%) neck pain, respectively. These results are similar with a study conducted by Hassanzadeh regarding the risk factors causing MSDs among a speed boat crew. Aghilinezhad et al. (2008) studied musculoskeletal problems among aircraft pilots, and reported the back area as the most painful zone with an incidence rate of 42% among helicopter pilots, and 40% among airplane pilots (13).

Statistical evaluation showed a significant relationship between the level of mental health with total job experience, working experience on speed boat, and hours spent on the boat per week in our study. This study revealed a close relationship between levels of mental health with designed variables; therefore we can improve the overall level of health by weight loss, appropriate exercises and decreasing work hours per week among this group of people. A limitation of this study was its cross-sectional design, small sample size and local military population. In addition, the use of self-administered questionnaires was other limitation. Another limitation is not considering other factors correlated with musculoskeletal complaints and mental health status such as different musculoskeletal structures and anxiety. To prevent musculoskeletal complaints, a multi-level approach is needed, including environmental measures and interventions directed to both psychosocial and organizational factors.
